# No obvious time trend in proximal humeral fracture complexity: a cohort study from 1944 to 2020 in Malmö, Sweden

**DOI:** 10.1302/2633-1462.69.BJO-2025-0210

**Published:** 2025-09-01

**Authors:** Anton Cederwall, Anders Nordqvist, Magnus K. Karlsson, Björn E. Rosengren

**Affiliations:** 1 Clinical and Molecular Osteoporosis Research Unit, Departments of Orthopedics and Clinical Sciences, Skåne University Hospital Malmo and Lund University, Malmö, Sweden

**Keywords:** Proximal humeral fracture, Epidemiology, Time trend, Incidence, Classification, Neer, AO, Radiographs, epidemiology, acute fractures, complex fractures, trauma, shoulder, reversed shoulder arthroplasty, shoulder dislocation, high-energy trauma

## Abstract

**Aims:**

The epidemiology of proximal humerus fractures (PHFs) has been described in terms of incidence, fracture complexity, and general time trends, but current literature on time trends in PHF complexity is limited. This study aims to explore possible time trends in PHF complexity and report the distribution of different types of PHF from January 1944 to December 2020.

**Methods:**

The city of Malmö, Sweden, has one emergency hospital where acute fractures are treated, and radiographs have been saved for almost a century. One author reviewed and classified relevant radiological examinations in individuals aged ≥ 18 years with a PHF during 17 sample years from 1944 to 2020 in Malmö using the Neer and AO classifications.

**Results:**

Of the 3,031 identified PHFs, 2,216 (73%) were sustained by women (mean age 69 years (SD 14)) and 815 (27%) by men (mean age 59 years (SD 17)). We saw no obvious time trend in fracture complexity overall, for men and women separately, or for different age groups. Fracture complexity according to AO was higher in older than younger age groups, which was true also with the Neer classification for women. However, for men, according to the Neer classification, the fracture complexity was higher in younger than older age groups.

**Conclusion:**

We found no obvious time trend in fracture complexity with the Neer or AO classification systems from 1944 to 2020.

Cite this article: *Bone Jt Open* 2025;6(9):1006–1012.

## Introduction

This is an extension of a previous study where we reported time trends in proximal humeral fracture (PHF) incidence.^1^ We found that 73% of the fractures occurred in women (mean age at fracture 69 years (SD 14)) and 27% in men (mean age 59 years (SD 17)).^1^

Previous studies have explored PHF epidemiology in terms of incidence^1-6^ and fracture complexity,^7-12^ but current literature on time trends in PHF complexity is limited. Bengnér et al^13^ noted a slight increase in complex fractures in Malmö by the Neer classification,^14^ with 73% Neer 1 (n = 384) in the 1980s compared to 80% Neer 1 (n = 329) in the 1950s. Regarding the AO classification,^15^ we have not been able to find any prior studies exploring temporal trends in PHF complexity.

The proportion of individuals treated operatively due to a PHF has increased during the first decade of the 21st century.^16-18^ This is possibly explained by an increase in PHF complexity, but whether PHF complexity has changed during this time-period is presently unknown.

The primary aim of this study was to explore possible time trends in PHF complexity between 1944 and 2020 using the AO and Neer classification systems. Second, we aimed to describe PHF complexity in relation to age and sex.

## Methods

Malmö is a city in the south of Sweden that had 131,718 adult inhabitants in 1944, which increased to 273,455 in 2020. The city has one emergency hospital, the Skåne University Hospital (SUS), where acute fractures were treated throughout the study period. Referrals, reports, and radiographs have been saved in the hospital archives since almost a century, and it is therefore possible to identify persons that have suffered a fracture during this time. In the current study the first author classified PHF from the previous study^1^ according to the Neer and AO classifications.

The Neer classification, devised by Charles S Neer II in 1970, divides the proximal humerus into four main parts: 1) humeral head; 2) greater tuberosity; 3) lesser tuberosity; and 4) shaft. Fractures with parts displaced less than 1.0 cm or angulated less than 45° are, regardless of the level or number of fracture lines, considered minimally displaced and classified as Neer 1. If displacement criteria are met for any fragment, the fracture is classified into one of the other subgroups. The subgroups are stratified into four types: Neer 1 (non/minimally displaced); Neer 2 (displaced two-part fracture); Neer 3 (displaced three-part fracture); and Neer 4 (articular or four-part fracture).^14^

The AO classification, initially published in 1987,^19^ subdivides PHF into 13 subgroups, stratified into three types: A (extraarticular, unifocal, two-part); B (extraarticular, bifocal, three-part); and C (articular or four-part).^15^

From 1944 to the turn of the millennium, both in- and outpatient radiological examinations performed at the SUS were archived in an analogue archive and subsequently in a digital archive. We reviewed the earliest and latest years available in both archives. Additional years were selected for uniform distribution. Radiographs from January 1996 to December 2000 were unavailable due to flood damage. Demographic variables (age, sex, fracture side) were recorded for everyone.

The first author (AC) reviewed all radiographs with the code S.F and Brach.F (i.e. a fracture in the shoulder- and brachialis region) during the years 1944 to 1946, 1952, 1957, 1962, 1967, 1972, 1977, 1981, 1987, 1994, and 1995. For the years 2005, 2010, 2015, and 2020, we searched in- and outpatient hospital records for the International Classification of Diseases 10^20^ code S42.2, (i.e. fracture of the upper end of the humerus). Radiographs of identified persons were retrieved by use of the unique personal identity number and then classified by the first author using Sectra IDS7 v. 24.1 (Sectra, Sweden). Only plain radiographs were reviewed. Malmö residents who suffered a PHF outside Malmö and attended a follow-up visit at SUS were included, in the same way as in previous similar studies from our region.^21^ The classification was then determined based on the follow-up radiographical examination.

A PHF was defined as a fracture within the proximal humeral region. The proximal humeral region was defined as the area created by a square with sides equal to the caput humeri width.^15^

For each classification system, we used established overarching types to permit a more synoptic presentation (i.e. Neer 1 to 4^14^ and AO A to C).^15^

The least complex fractures are classified as Neer 1 in the Neer classification, and as type A in the AO classification. The most complex fractures are classified as Neer 4 in the Neer classification and as type C in the AO system. We use the term higher fracture complexity to describe a higher proportion of fractures classified as Neer 3 or 4 and/or AO types B or C.

To evaluate interobserver agreement, two authors (an orthopaedic consultant with a subspeciality in shoulder surgery since 2018 (AC) and one senior shoulder surgeon (AN)) classified 40 consecutive PHFs using the Neer and AO classifications. The number of fractures was determined from previous similar agreement calculations.^22,23^ To evaluate intraobserver agreement, AC repeated the classification of the same fractures six weeks later. The agreement, described as percent (Cohen’s kappa (κ)),^24^ was in terms of interobserver agreement for Neer 90% (0.76) and for AO 85% (0.77) and in terms of intraobserver agreement for Neer 93% (0.81) and for AO 93% (0.89).

Ethical approval for the study was obtained prior to study start from the regional ethical review board of Lund University (LU 2012-394).

### Statistical analysis

We used SPSS v. 28.0 (IBM SPSS, USA) and Excel v. 16.67 (Microsoft, USA) for database management. Due to the long-term study design and the relatively low number of fractures during each timepoint, we used descriptive rather than inferential statistics. Trends were evaluated by visual inspection.

## Results

Overall, 2,182 (72%) of the 3,031 fractures were classified as Neer 1 and 1,748 (58%) as AO type A. Detailed distribution regarding overarching types and sub-groups are presented in Table I and sex-specific data is presented in Supplementary Table I.

**Table I.** Distribution of proximal humeral fractures and mean age per overarching type by the Neer and AO classification in 3,031 Malmö residents aged ≥ 18 years (2,216 women with a mean age of 69 years (SD 14) and 815 men with a mean age of 59 years (SD 17)), during 17 sample years from 1944 to 2020.

Neer			AO		
Type/Subgroup	N (%)	Mean age, yrs (SD)	Type/Subgroup	N (%)	Mean age, yrs (SD)
Neer 1*	2,181 (72.0)	66 (16)	A†	1,748 (57.7)	64 (17)
Neer 2*	561 (18.5)	68 (16)	B†	935 (30.8)	70 (13)
Neer 3*	163 (5.4)	71 (12)	C†	348 (11.5)	70 (12)
Neer 4*	126 (4.2)	71 (12)			
I	2,181 (72.0)	66 (16)	A1.1	614 (20.3)	55 (17)
II	4 (0.1)	77 (10)	A1.2	27 (0.9)	60 (21)
III	346 (11.4)	71 (14)	A2.1	931 (30.7)	70 (15)
IV	78 (2.6)	68 (16)	A2.2	13 (0.4)	77 (13)
V	10 (0.3)	60 (17)	A2.3	152 (5.0)	68 (16)
VI	113 (3.7)	60 (17)	A3	11 (0.4)	66 (15)
VII	10 (0.3)	56 (18)	B1.1	903 (29.8)	70 (13)
VIII	133 (4.4)	72 (12)	B1.2	32 (1.1)	67 (13)
IX	11 (0.4)	71 (10)	C1.1	196 (6.5)	71 (12)
X	16 (0.5)	64 (11)	C1.3	14 (0.5)	76 (9)
XI	3 (0.1)	46 (16)	C3.1	94 (3.1)	69 (14)
XII	78 (2.6)	70 (12)	C3.2	41 (1.4)	71 (12)
XIII	20 (0.7)	74 (10)	C3.3	3 (0.1)	70 (13)
XIV	3 (0.1)	57 (13)			
XV	0 (0)	N/A			
XVI	1 (<0)	57 (N/A)			
XVII	24 (0.8)	74 (11)			

* Neer 1, minimally displaced; Neer 2, displaced two-part; Neer 3, displaced three-part; Neer 4, displaced four-part or articular surface.^14^

† A, Extraarticular, unifocal two-part; B, Extraarticular, bifocal, three-part; C: Articular or four-part.^15^

N/A, not applicable.

### Trends in fracture complexity

**Fig. 1:**
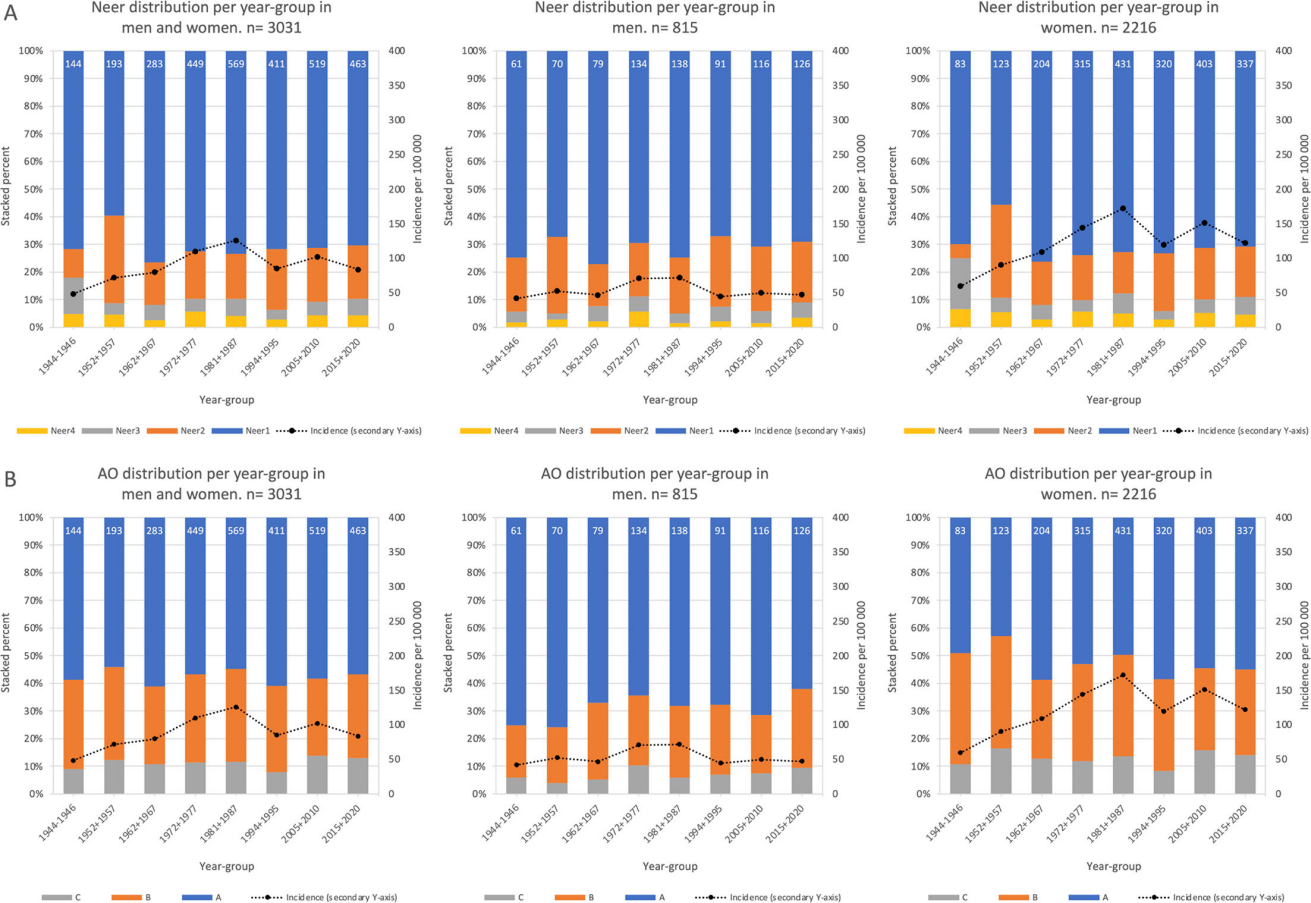
Fracture distribution and incidence rates per year-group for proximal humeral fractures in Malmö residents aged ≥ 18 years during 17 sample years from 1944 to 2020. On the primary y-axis, fracture distribution based on incidence is presented as stacked percent per fracture subgroup while incidence rates per 100,000 person years are presented on the secondary y axis. Data are sex- and age-adjusted for the entire cohort (left) and age-adjusted for men (middle) and women (right). Panel A depicts classification according to Neer, and B according to AO. Data labels represent fracture numbers.

We did not observe any obvious time trends in fracture complexity (regardless of classification system or if we included all data or stratified by sex and age (Figure 1, Supplementary Figures Ia to b, and Supplementary Figures IIa to c).

**Fig. 2:**
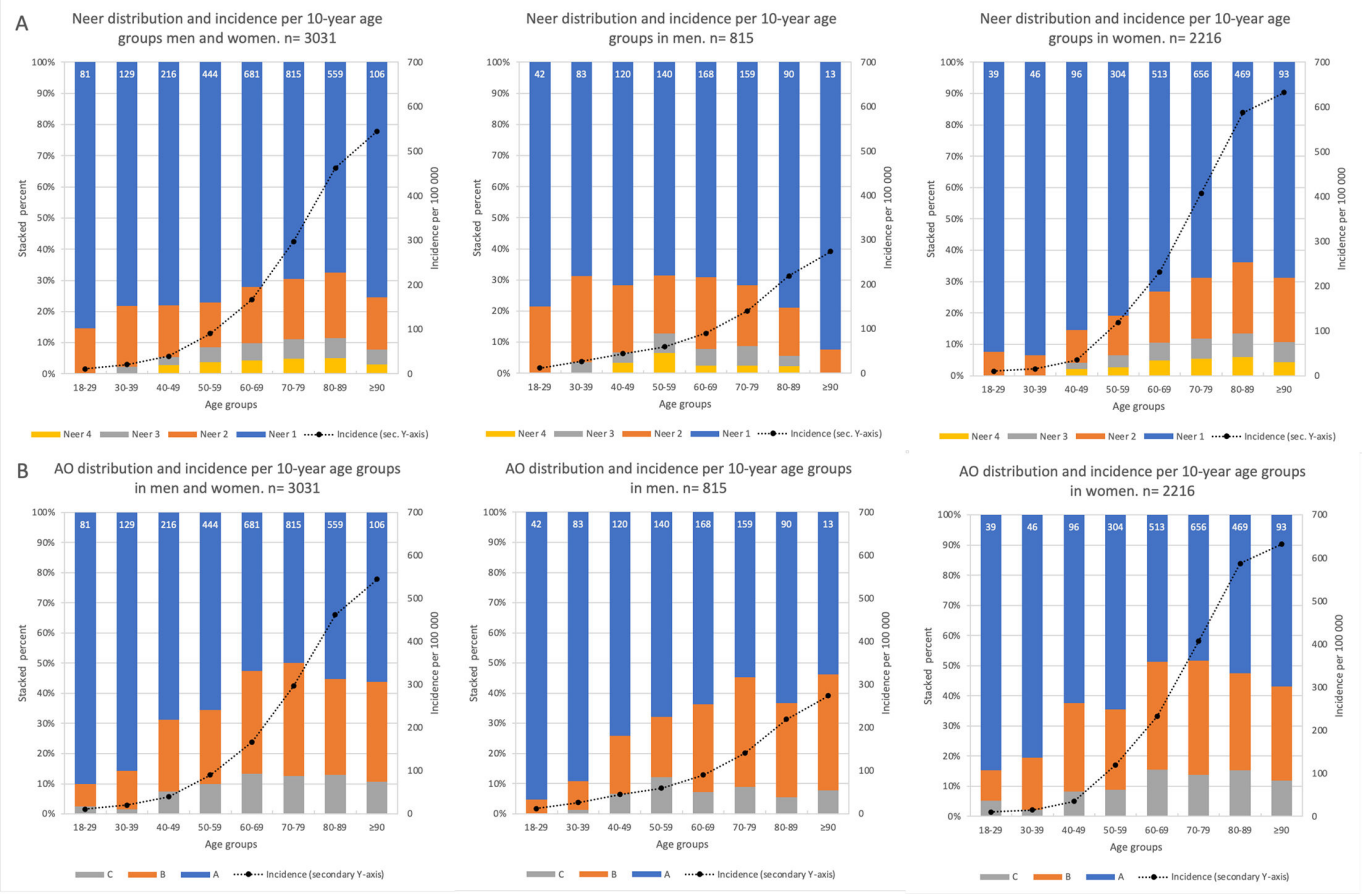
Fracture distribution and incidence rates per age group for proximal humeral fractures in Malmö residents aged ≥ 18 years during 17 sample years from 1944 to 2020. On the primary y-axis, fracture distribution based on incidence is presented as stacked percent per fracture subgroup while incidence rates per 100,000 person years are presented on the secondary y-axis. Data are sex-adjusted for the entire cohort (left). Panel A depicts classification according to Neer and B according to AO. Data labels represent fracture numbers.

Fracture complexity according to AO was higher in older than younger age groups, which was evident also with the Neer classification for women. For men according to the Neer classification however, fracture complexity was higher in younger than older age groups (Figure 2). Data on fracture subgroup-specific incidence in relation to age for the two classification systems are presented in the supplement (Supplementary figure III).

## Discussion

In this extension of our previous study of PHF occurrence,^1^ we did not observe any obvious time trends in fracture complexity from the 1940 s until 2020 with the Neer or AO classification systems. We found that the age trend in fracture complexity depended on classification system and sex. Higher age seemed associated with higher fracture complexity for both men and women according to the AO classification, as well as for women according to the Neer classification. For men according to the Neer classification however, younger age seemed associated with higher fracture complexity.

The current literature on time trends in PHF complexity is scarce. Bengner et al^13^ described a “slight tendency” towards more complex fractures in Malmö in the 1980s (73% Neer 1 (n = 384)) compared to the 1950s (80% Neer 1 (n = 329)). Hypothetically, PHF complexity could have increased over time, since the average age^25^ and the frequency of osteoporosis^26^ is higher today compared to the 1940s. Our finding of a similar fracture complexity from the 1940s to 2020 has, to our knowledge, not previously been reported. While an increase in operative treatment of PHFs during the first decade of the 21^st^ century has been documented in prior studies,^17,18,27^ the underlying drivers of this change are unknown. Since fracture complexity seems stable over time, other causes such as changes in indications and introduction of new technologies^17,18^ (locking plates, reversed shoulder arthroplasty) could be possible explanations for the increase in operative treatment.

The mean age and men:women ratio in the current study was 3:7, similar to a Scottish study from 2001.^8^ The authors, however, reported 49% Neer 1 (in 1,027 PHFs), whereas we found 72% (Table I). A weakness in the Scottish study is that data on intra- or interobserver agreement were not presented.^8^ Neer, in his original study from 1970, reported 85% Neer 1 fractures,^14^ but subsequent epidemiological studies^9,11,12^ (Supplementary Table II) have not been able to confirm this high proportion,^14^ and the figures have recently been questioned.^28^ Previous studies have reported an interobserver agreement of the Neer classification between fair (κ 0.21 to 0.40) to moderate (κ 0.41 to 0.60).^22,23,29^ We found a substantial interobserver agreement (κ = 0.76). The large variation in interobserver agreement between studies may contribute to the apparent differences in the proportion of Neer 1 fractures as may differences in year of data collection, population of examination, and geographical area.

The Scottish study^8^ also reported the fracture distribution by the AO classification with 66% type A, 27% type B, and 6% type C fractures. Corresponding figures from another Swedish study examining year 2011 to 2013 (in 1,582 PHF), were 45% type A, 44% type B, and 11% type C fractures,^7^ while a German study^10^ examining year 2006 to 2011 (in 815 PHF) found 46% type A, 22% type B and 32% type C fractures. The high proportion of type C fractures in the German study may be attributed to its setting in a level 1 trauma centre since 183 (22%), presumably complex fractures, of the included patients were referred from other hospitals. In the current study, we found 58% type A, 31% type B, and 12% type C fractures (Supplementary Table II). The slight (κ = 0.11) interobserver agreement of the AO classification reported by Majed et al^30^ is a possible contributor to the found differences. We found a substantial interobserver agreement (κ = 0.77) while several studies^7,8^ do not report the intra- or interobserver agreement. The Swedish study,^7^ based on the Swedish Fracture Register (SFR), is contrasted by the Scottish study,^8^ as well as the current study, in which all fractures were classified by the same physician. Varying level of experience of classifiers between studies could thus have contributed to the different results. Complex fractures may also be more likely to be registered in the SFR, as the chance of registration probably increases with more follow-up visits and more operative procedures. This may also have contributed to the higher proportion of type B than type A fractures in the Swedish study compared to the current and other studies.^8^

Fracture classification distribution in previous studies from Scotland,^8^ Japan,^9^ Germany,^10^ France,^12^ Sweden,^7^ and Spain^11^ in relation to the findings from the current study is presented in chronological order in Supplementary Table II. Compared to these studies, the fractures in the current study were generally of lower complexity according to the Neer classification and of similar complexity according to the AO classification.^7-12^

Higher age seemed associated with higher fracture complexity for both men and women according to the AO classification, as well as for women according to the Neer classification. This is in line with previous studies.^11,12,31^ Explanations for this may include a more fragile skeleton and an inferior ability to parry a fall at higher than younger age, resulting in more complex fractures. For the Neer classification in men we found, however, that younger age seemed associated with higher fracture complexity, in line with results from Germany.^31^ This seems partly driven by Neer group VI (i.e. isolated greater tuberosity fractures with concomitant shoulder dislocation), that stands out in men aged < 50 years (11%) in a secondary review of our material compared to all individuals with a PHF (4%). Passaretti et al^32^ reported on the PHF trauma mechanism in Rome, Italy, between 2013 and 2016 (in 711 PHFs), and found a dominance of men in sports- and high energy-trauma and a dominance of women in low-energy trauma. We therefore speculate that men tend to suffer more complex fractures, especially Neer VI, at younger age, possibly due to high-energy trauma, while women may rather suffer more complex fractures at older age, largely related to low-energy trauma. A possible explanation for why women sustain more complex fractures at older ages is a more fragile skeleton (i.e. osteoporosis), which is more common in older than younger women.^33^

Our inter- and intraobserver agreement is higher than most prior studies.^22,23,34^ Sidor et al^23^, however, reported an intraobserver agreement for Neer classification by a shoulder specialist similar to ours. Brorson et al^35^ also demonstrated that educating a group in the Neer classification improved the interobserver agreement from fair to substantial. Part of the explanation to our high agreement might be that the first author (AC) is a shoulder specialist and was also specifically trained in PHF classification prior to study start by a senior expert (AN).

Strengths of the current study include the study period of almost 80 years, since time trend data for fracture complexity is relatively limited and short-term in the present literature. Additionally, the fact that the same shoulder surgeon reviewed and classified all fractures, with high intra- and interobserver agreements, should also be regarded as a strength.

The findings of this study may not be generalizable to very different settings. However, given Sweden’s high fracture incidence,^36^ our results might provide valuable insights into fracture epidemiology and enable detection of emerging trends.

Study limitations include the use of two case-finding strategies, one in the analogue and another in the digital archive. The analogue archive is not validated, as no obvious gold standard comparator is available. However, we observed examinations in the analogue archive from the beginning of January until the end of December in every examined year, as well as consecutive label numbers. The case finding strategy in the digital archive has previously been validated in our setting, with a misclassification rate of about 3%, leading to a possible small underestimation of the true incidence.^21^ Another weakness is that we, due to constraints in time and resources, did not analyze every year of the study period. Additionally, some individuals with a PHF who did not reside in Malmö were probably included if they underwent radiological examinations there between 1944 and 1995. However, data from 1981 (where more comprehensive individual information is available), indicate that only 1.0% of PHF patients lived outside Malmö, suggesting only a possible minor overestimation in earlier years. Conversely, Malmö residents who sustained a PHF elsewhere are likely included due to follow-up visits and radiological exams conducted upon their return. It would have been advantageous with an even larger study, including more fractures, especially for unusual fracture types and for smaller sex-specific age groups.

In conclusion, we saw no obvious time trend in fracture complexity with the Neer or AO classification systems between 1944 to 2020 in Malmö, Sweden. Fracture complexity according to AO was higher in older than younger age groups, which was true also with the Neer classification for women. For men according to the Neer classification however, fracture complexity was higher in younger than older age groups. The increase in operatively treated PHFs during the first decades of the 21st century is thus probably attributed to changes in other factors than fracture complexity.

**Take home message**

- We saw no obvious time trend in fracture complexity with the Neer or AO classification systems during the more than 70-year study period.

- The increase in operatively treated proximal humerus fractures during the first decades of the 21st century is probably attributed to changes in factors other than fracture complexity.

## Data Availability

The data that support the findings for this study are available to other researchers from the corresponding author upon reasonable request.
